# Off-label drug use in palliative medicine: Delphi study for the consensus of evidence-based treatment recommendations

**DOI:** 10.1177/02692163251323123

**Published:** 2025-03-15

**Authors:** Stefanie Pügge, Aleksandra Dukic-Ott, Julian Baumgärtel, Saskia Jünger, Claudia Bausewein, Constanze Rémi

**Affiliations:** 1 Department of Palliative Medicine, LMU University Hospital, Ludwig-Maximilians-University in Munich, Munich, Germany; 2 Department of Health Sciences, Hochschule Bochum, Bochum, Germany

**Keywords:** Off-label use, palliative medicine, Delphi technique, patient safety, drug information services

## Abstract

**Background:**

Off-label use of drugs is an integral part of everyday clinical practice in palliative medicine. However, it is associated with many uncertainties, that is, drug therapy safety or legal issues including cost coverage. Healthcare professionals often lack time and resources for comprehensive literature search and patient-specific risk-benefit analyses.

**Aim:**

The aim of this project is to develop, evaluate and rate agreement/disagreement on treatment recommendations for off-label use in adult palliative medicine.

**Design:**

Online Delphi study with two rounds each to rate agreement/disagreement with treatment recommendations for off-label use in adult palliative medicine. An international expert panel consisting of physicians, pharmacists and nurses working in palliative care evaluated previously developed recommendations based on the best available evidence.

**Setting:/participants:**

Professionals (physicians, pharmacists, nursing staff) working in inpatient and home palliative care involved in the medication process were recruited as experts to participate. Between 64 and 75 experts participated in the first two Delphi studies.

**Results:**

A total of 64/68 recommendations on 21 drugs and 14 applications were agreed upon. Topics related to routes of administration as well as indications for sialorrhea, bronchorrhea, xerostomia, pruritus, singultus, fistula, gastroparesis and hot flashes. Recommendations that reached consensus are available to health care professionals via a free of charge database.

**Conclusion:**

For many off-label use applications, it is likely that there will be no registration studies and therefore no drug approvals in the future. The consensus-based recommendations are intended to facilitate individual treatment planning for prescribers and to enable a more reflected handling of off-label use.

**What is already known about the topic**

- Off-label use of drugs for symptom management is part of everyday clinical practice in palliative medicine.
- Off-label use requires a thorough and individual risk-benefit-analysis to avoid patient harm.
- Lack of available evidence leads to experience-based decision making.

**What this paper adds**

- A standardised procedure enables the optimal integration of the best-available evidence and the widest clinical expertise on off-label drug use in palliative care.
- The resulting treatment recommendations allow clinicians to make a simple distinction between experimental off-label use and scientifically proven off-label use in palliative medicine.
- The treatment recommendations provide a reliable foundation for informed decision-making regarding pharmacotherapy for patients in the palliative care setting, supporting the delivery of safe and effective treatments.

**Implications for practice, theory or policy**

- The therapeutic spectrum might be broadened and valuable treatment options added for individual patients.
- Practitioners might be more willing to share their experiences on off-label use of drugs and make them available to a wider public.
- Hopefully, challenges associated with palliative care pharmacotherapy will be better appreciated by those involved in policy making. This is with a view to increasing support for research initiatives aimed at developing a more robust evidence base.

## Introduction

According to the definition of the European Medicines Agency (EMA), off-label use is the use of a medicine for an unapproved indication or in an unapproved age group, dosage or route of administration.^1^

Particularly in paediatrics,^2–4^ oncology^5,6^ and palliative medicine,^4,7–9^ off-label use of drugs is not uncommon. The reasons are manifold.^10,11^ A retrospective analysis of patient records in a palliative care unit in Germany demonstrated that almost 50% of medicines were used off-label. In the case of relevant frequently used drugs in palliative medicine like ketamine, haloperidol, hyoscine butylbromide and morphine, this was true for two thirds of applications.^12^

Palliative medicine aims to maintain or improve quality of life of people with advanced disease. The off-label use of drugs in this field is associated with many uncertainties in everyday life, such as drug therapy safety or legal aspects including cost coverage.^7^

For any drug therapy, potential risks should always be weighed against potential benefits; in palliative medicine, this is usually the relief of burdensome symptoms.^13^ The extended German Guideline ‘Palliative care for patients with incurable cancer’ contains numerous evidence- or consensus-based off-label recommendations on common symptoms.^14^ However, the guideline is limited to oncological patients and does not cover rare symptoms or alternative routes of administration. Other sources do not fill these information gaps, or do so inadequately. This is a challenge for healthcare professionals in daily clinical practice: There is a lack of time and resources to systematically search for and appraise the evidence to take optimal treatment decisions for individual patients.^15^ The scarcity of high-level evidence in palliative medicine is an additional factor.^16,17^ Randomised controlled trials (RCTs) are not always justifiable or possible for ethical, economic or practical reasons.

### Rationale

The off-label use of drugs is of cross-border relevance to palliative care. The focus is on the safety and effectiveness of the treatment, although financial aspects may be relevant in the national context. While clinical experience may differ in different parts of the world, the available scientific evidence is the same everywhere.

### Purpose

The aim of this study is to develop, evaluate and consent treatment recommendations for off-label use of drugs in adult palliative medicine. In particular, it is intended to provide prescribers and other healthcare professionals with an evidence-based decision-making tool for clinical practice where high-quality trials are lacking.

## Methods

### Design

Online Delphi study with two rounds each to rate agreement/disagreement with treatment recommendations for off-label use of drugs in adult palliative medicine. The study is reported according to the Guidance on Conducting and REporting DElphi Studies (CREDES) in palliative care.^18^

To achieve the long-term goal of the overall project^19^ of approximately 400 off-label use recommendations by 2028, these were divided into several Delphi studies, each consisting of 25–40 recommendations. The recommendations were grouped by indications and routes of administration. Each Delphi study is self-contained. This article deals with the first two Delphi studies focusing on off-label routes of administration as well as off-label indications for sialorrhea, bronchorrhea, xerostomia, pruritus, singultus, fistula, gastroparesis and hot flashes.

To address the challenges of conducting classical RCTs in the palliative care context, the implementation of other non-RCT study designs may be a better alternative.^20–22^ Expert opinion and clinical experience also play a key role in these cases, for example in the development of guidelines and treatment recommendations.^23^ In palliative care, the Delphi technique has been successfully used to collect, evaluate and reach consensus on recommendations and assessment tools.^18,24^ This multi-stage consensus process allows limited scientific evidence to be combined with empirical knowledge to generate statements. It is assumed that a group of experts with different perspectives will provide a more valid result than the opinion of a single expert, no matter how experienced that expert may be.^25^ In compliance with defined scientific standards and reporting criteria, a basis for decision-making is provided for clinical practice.^18^

### Procedure

**Figure 1.**
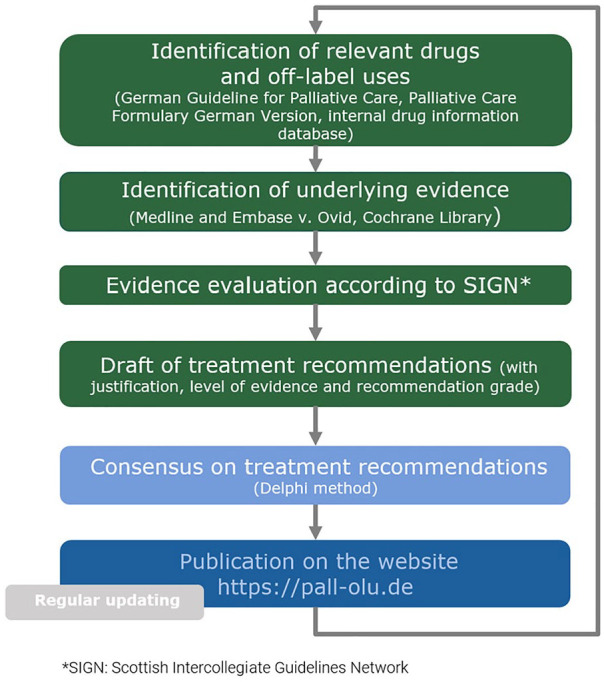
Development of treatment recommendations in accordance with the recommendations of the Group Delphi.^15^

The draft treatment recommendations were developed following a standardised procedure for selected drugs and indications commonly used in palliative medicine (see Figure 1).^15^ Selections of drugs was mainly based on the WHO Guideline on Palliative Care^26^ and the German Palliative Care Guideline.^14^ Furthermore, drugs and off-label indications mentioned in the German reference book ‘Arzneimitteltherapie in der Palliativmedizin’ (German version of the Palliative Care Formulary)^27^ and in the database of the department’s drug information service were considered. The recommendations were presented according to the German Palliative Care Guideline with levels of evidence and grades of recommendation.^28^

### Expert panel

Population: The expert panel for the Delphi studies consisted of palliative care professionals working in inpatient and home palliative care and involved in the palliative care medication process.

#### Inclusion criteria

- Physicians from different disciplines working in generalist or specialist palliative care
- Pharmacists regularly involved in the care of palliative patients
- Nursing staff with a focus on palliative medicine
- German speaking

Sampling and recruitment: Potential participants were initially identified and contacted through a core group of experts who had previously partipated in the development of the project^15^ using a snowball method.^29^ Further individuals with expertise in palliative drug therapy were identified and contacted via the online portal Hospice and Palliative Care Guide for Germany.^30^ Between the first and the second Delphi study, further experts were recruited for participation to ensure a high potential number of participants. A certain degree of fluctuation is always to be expected due to higher workloads, professional reorientation and holiday periods.

To ensure the recommended number of at least 10–50 participants in each survey round,^29^ a total of approximately 100 people were recruited from the above mentioned groups in three German-speaking countries (Germany, Austria, Switzerland) because the available drugs used in palliative care in these countries are very similar. This allowed for a sufficiently large base of different perspectives and experiences to reach consensus.^25^ For the second round of the Delphi study, those experts who had not participated in the first round were contacted again. As an incentive for participation, two text books (1st Delphi study) or online participation in a workshop on palliative care pharmacotherapy (2nd Delphi study) were raffled off among all those who completed both rounds of the survey. Participation in the Delphi study was voluntary. Written information about the study details was provided to participants via e-mail.

### Study conduct

Consensus was reached through a structured Delphi study with two rounds, followed by a final expert consultation. The questionnaire for the first round consisted of recommendations to be agreed upon, grouped by indication or route of administration, and demographic data. All participants received a summary of results of the literature review in form of evidence tables. To test applicability of the questionnaire, each round was piloted with five palliative medicine physicians, pharmacists and nurses from different care settings. The online questionnaire was finalised, sent and administered using the software Lime Survey.^31^

The estimated time required to complete the questionnaire for the first round of the survey was approximately 30–45 min. Submission was only possible after answering all mandatory questions. Predefined response options were single choice, multiple choice and free text.

Respondents were asked to rate their level of agreement with each statement on a five-point Likert scale (5 = strongly disagree, 4 = somewhat disagree, 3 = neither agree or disagree, 2 = somewhat agree, 1 = strongly agree). In addition, a question was asked about the certainty of the response to each statement (very certain, slightly certain, slightly uncertain, very uncertain). This served as a filter for the evaluation. Only the very certain and slightly certain responses were considered for consensus. This gave participants the opportunity to assess their own competence in evaluating each recommendation. To allow participants to abstain from voting, the option ‘no response’ was introduced in the second Delphi study to allow biased or prejudiced participants to abstain from individual recommendations. Under each statement there was space for free text comments, for example, revisions to the wording or explanations of the assessment. Open comments were analysed by the project team after each round and recommendations revised or reformulated where necessary. In case of major uncertainties, the expert panel could also be consulted.

**Figure 2.**
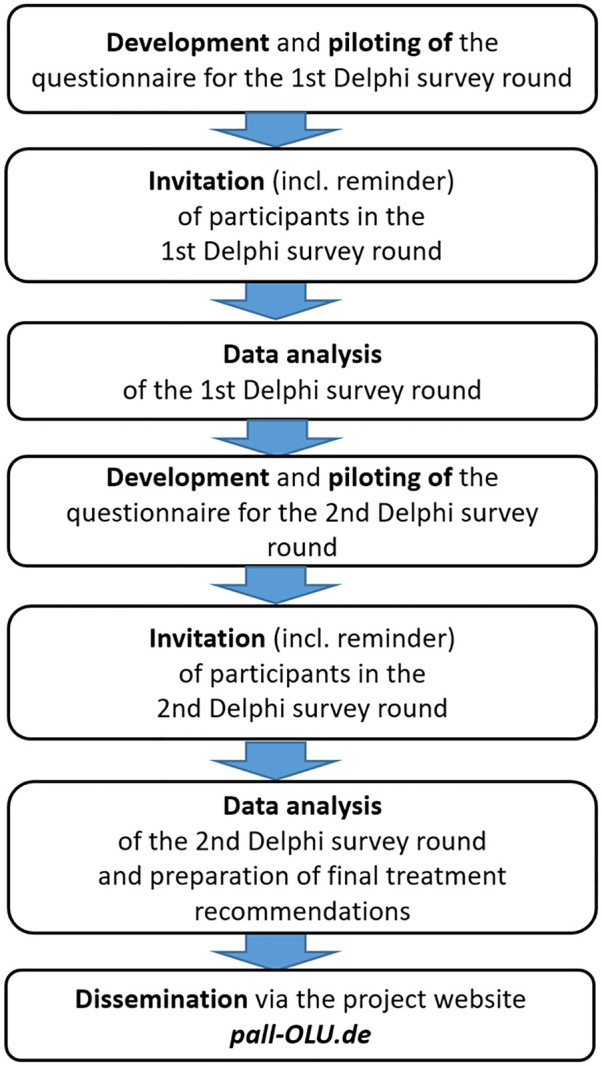
Stages of the Delphi studies.

Each Delphi study was divided into several stages (see Figure 2).

The invitation to the surveys was sent to participants via email. The questionnaire was accessible for 3 weeks plus an optional one-week extension, with participation reminders after 10 days and 3 weeks. At the end of Round 1, responses and free text comments were analysed anonymously. Round 2 of the Delphi study contained all statements that did not reach consensus in Round 1 or that required a revision of the content. The participants were provided information on percentage of consensus and certainty of statements of the first round that did not reach consensus.

### Definition and attainment of consensus

Consensus was reached when at least 80% of the experts who voted agreed very strongly or somewhat strongly (strongly agree, somewhat agree). As there is no set standard for consensus definition in Delphi studies^32,33^ the threshold of 80% for consensus was chosen, as described in other studies^34^ to ensure recommendations are based on the opinion and experience of the large majority of experts.

Descriptive statistics after each round included information on consensus (percentage agreement, median and interquartile range, IQR) and certainty. For each statement, the free text comments were entered into a table. This was discussed by the study group to identify semantic, practical and conceptual problems. Based on the qualitative analysis of the comments, treatment recommendations were rephrased or deleted. At the end of Round 2, responses and free text comments were analysed and all agreed treatment recommendations were compiled. The statistical analysis was conducted using SPSS version 29.

### Publication and dissemination

Consented recommendations were published on the project website pall-olu.de.

### Ethics approval

The study was approved by the Local Research Ethics Committee at the Medical Faculty of Ludwig-Maximilians-Universität München (project no. 22-0252/4.5.2022).

## Results

For the first Delphi study in May and July 2022, of 104 people who were invited to participate in both rounds, 64 took part in the first round (response rate 61.6%) and 67 in the second round (response rate 67.9%).

In the second Delphi study in March and May 2023, respectively, 75/132 experts contacted took part (response rate: 56.8%) and 60/130 in the second round (response rate: 46.2%).

Each Delphi study covered different topics and recommendations (Table 2).

Invitation links that could not be delivered were sent again individually via the standard mail programme.

Table 1 summarises the demographic data of participants in both Delphi studies. The majority of the expert panel consisted of physicians, with the majority of respondents coming from Germany. Over 80% of the participating expert panel had at least 5 years of professional experience in palliative medicine.

**Table 1.** Demographics of participants of the two Delphi studies.

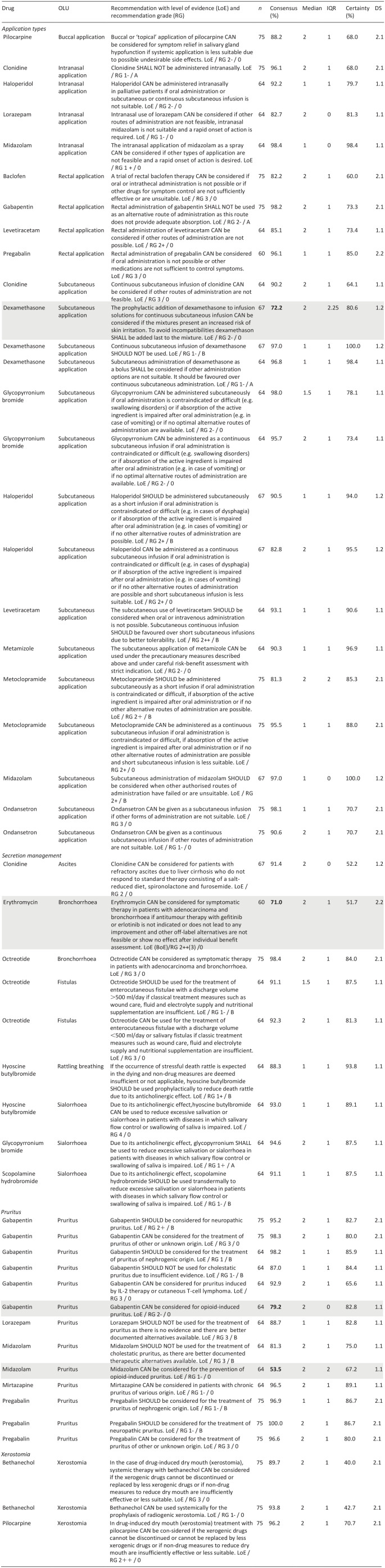

* Fields highlighted in grey were not queried in the 1st Delphi study.

**Figure 3.**
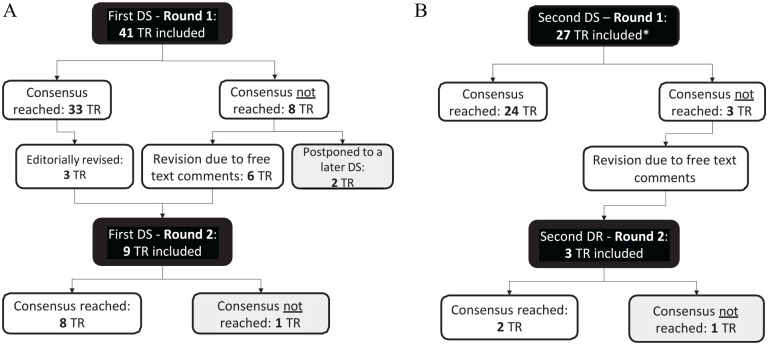
Flow diagram of the two-stage Delphi study: (A) First Delphi study, (B) Second Delphi study. DS: delphi study; TR: therapy recommendation. *2 recommendations on pruritus were adapted again after the first DS based on the S2k guideline on pruritus and included again in the 2nd DS.

A total of 64 out of 68 recommendations on 21 active substances and 14 applications reached consensus in both Delphi studies. In the first study, three of the recommendations already approved in the first round were revised based on the participants’ feedback and put to vote again. Two non-consented recommendations on the topic of opioid-induced pruritus were postponed after the first round of the first Delphi study to set a thematical focus on opioid-induced pruritus in a later Delphi study. One treatment recommendation in each of the Delphi studies was not consented (Figure 3).

The non-consented recommendations (dexamethasone subcutaneously as a prophylactic addition in continuous subcutaneous infusions, erythromycin for bronchorrhoea) were discussed again in an expert workshop on off-label use in January 2024. It was decided that non-consented recommendations will not be published on the website pall-OLU.de but might be reconsidered for a Delphi study at a later stage if new evidence is available.

Table 2 lists recommendations for types of off-label application, secretion management and xerostomia (incl. text, grade of recommendation, level of evidence according to SIGN (Scottish intercollegiate guidelines network)) with consensus and certainty. Further recommendations regarding gastroparesis, hiccups and hot flushes as well as changes to the recommendations between the first and second round of the respective Delphi studies are listed in the Supplemental Material.

**Table 2.** Selected consented and not consented treatment recommendations of the first two Delphi studies.

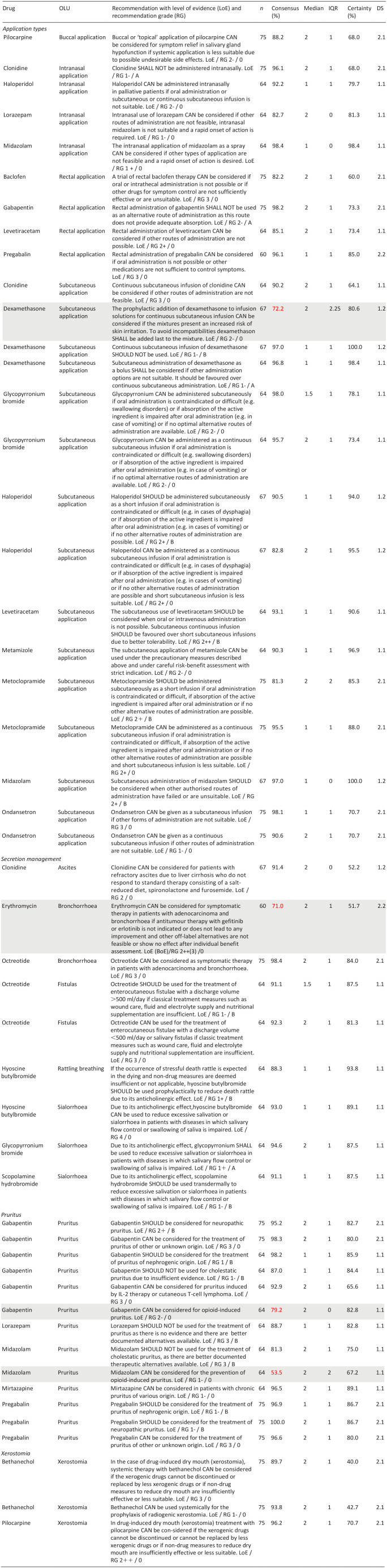

*n*: participants in the respective Delphi procedure; consensus: % agreement of very certain/slightly certain answers; certainty: % votes with very certain/slightly certain; IQR: interquartile range; DS: consensus achieved in Delphi study and round X.Y.

Not consented treatment recommendations are highlighted in grey; bold numbers indicate non-achievement of consensus.

## Discussion

Off-label use of drugs is part of everyday clinical practice in palliative medicine.^11,35^ In the project described here, for the first time, recommendations for off-label use in the context of adult palliative medicine were developed based on available evidence, evaluated, rated and published for the German-speaking countries. The study was focused on German-speaking countries because the available drugs used in palliative care are very similar. Moreover, we wanted to reach consensus on the treatment recommendations in the language in which they will ultimately be made available to minimise linguistic misunderstandings. Since the underlying data of the treatment recommendations are international data, the transferability to other countries is basically given. As the available scientific evidence applies to the international context, recommendations might be translated and transferred to other countries, although the legal status and availability of drugs might differ. The Delphi technique has previously been used to reach consensus on treatment recommendations in the geriatric context and for specific indications.^36–38^ Similarly, consensus building through Delphi studies is now well established in palliative care.^39–41^

Recruiting various professional groups from different settings allows for multiple perspectives and knowledge to be included.^42^ This approach has several advantages: the anonymised individual survey avoids potential influences of group conformity or responses from others. Respondents can change their opinion between rounds (iteration) and receive feedback in the second round on the non-consented results of the previous round. Though we invited more experts to participate in the second Delphy study, the response rate was lower for unknown reasons. In absolute figures, the number of participants was not that different between the two rounds. We see two possible reasons for the decrease in the response rate: first, curiosity was no longer as high in the second round and second, we did not ask who was willing to take part before sending out the invitation.

Most abstentions were recorded for the recommendations on bethanechol for xerostomia and clonidine for gastroparesis, as well as for intranasal clonidine, subcutaneous ondansetron and erythromycin for bronchorrhoea. The greatest uncertainties also occurred with these recommendations. Therefore, it is possible that the lack of experience may have contributed to the decision to abstain. In case of recommendations for less common applications or indications, such as hot flushes or bronchorrhoea, there may be few experts, even among the experienced ones, who take part in the vote on the topic in question. This is reflected in a lower percentage of certainty. It may therefore be necessary in the future to involve other specialists for certain topics, for example, with more expertise from an oncological or neurological context.

### Implications for practice

In clinical practice, there is a lack of resources for searching and assessing the evidence for off-label uses.^7,43^ The available recommendations summarise the limited evidence base in the first place and combine it with the expert experience. This facilitates individual therapy decisions based on the best available evidence and hopefully increases the likelihood of a positive risk-benefit balance for the patient. The consented recommendations, including background information for healthcare professionals, are available free of charge on the website https://pall-OLU.de. To embed the treatment in an overall context, the database with the treatment recommendations also includes instructions for use, monitoring parameters for therapies, alternative therapeutic options and information on the approved indications of the respective drugs. Information on the safety of drug therapy or formulations is also listed. As the evidence base can change over time, it is planned to regularly update both the consented and non-consented recommendations. This will include the inclusion of new data into the background texts and, if necessary, renewed consensus if the level of recommendation changes. To respond to the needs of users, a survey on the use of the database is planned for 2025.

A challenge for such a project is the lack of evidence of drug treatments in the palliative care population. Thus, recommendations are partly derived from publications with low level of evidence, such as case reports or case series, as no further publications are available. At the same time, this is the aim of the project: to provide the best possible assessment and recommendations in cases where high-quality data are lacking.

### Implications for research

Throughout searching and evaluating the evidence we identified multiple knowledge gaps with very limited evidence indicating where further research is needed. A possible approach to highlight important areas for research could be a ranking system of the gaps. Furthermore, the publication of more high quality data from palliative care studies is emphasised as well as publications of clinical experiences to share knowledge with other health care professionals. During the next 4 years of this project, it is planned to develop, evaluate and publish approximately 400 recommendations in the database to cover other relevant topics in palliative care such as off-label use of drugs for gastrointestinal, neuropsychiatric, respiratory or pain-related symptoms as well as further routes of administration. This will provide a comprehensive database of recommendations to support treatment recommendations for palliative care professionals.

### Strenghths and limitations

One of the strengths of the studies described here is the high level of participation in the voting and the participation of a large number of experts from different fields with long-standing professional experience. In each round, more than 60% of the participants had more than 15 years of general professional experience. More than half to almost two thirds of the participating physicians work in an internal medicine and/or palliative medicine setting. More than three fourths have also completed further training in palliative care. At the same time, participants were given the opportunity to assess their own experience by indicating their level of certainty. Obviously, there is no absolute certainty for the recommendations, that are transparent assessments by experts made to the best of their knowledge and belief.^44,45^ This was further supported by the introduction of the ‘no response’ option in the second Delphi study. This allowed biased or prejudiced participants to specifically abstain from individual recommendations.

A limitation is the focus on German-speaking countries as recommendations are currently only available in the German language. As the literature search is based on international evidence it is possible to translate and transfer recommendations to other country settings in most cases. Further publications in English are also planned for the future to make recommendations accessible.

Another limitation is, that for technical reasons, only fully completed questionnaires could be analysed in an anonymous survey. In the case of incomplete questionnaires, it was possible for the same person to vote more than once, so that the answers were potentially included twice in the analysis. However, votes may also have been lost in this way. As this limitation only became apparent in the second Delphi study, the results of the first study were re-analysed. This did not change whether or not consensus was reached.

As it is not necessary to have participated in the first round to vote in the second round, it is also possible that people with different opinions or certainty voted in the two rounds. This could affect the consensus. To minimise the influence of this potential limitation, incentives were created to encourage participation in both rounds of the Delphi study.

## Conclusion

For many drug applications in symptom management, it is likely that there will be no authorisation studies and therefore no marketing authorisations in the future. With the publication of recommendations, the project pall-OLU.de provides healthcare professionals for the first time with a free database with specific information on the off-label use of drugs in palliative medicine. The goal is to support prescribers in planning treatments and decide in favour of or against treatment options in an individual context. Additional recommendations and information will be added over the next 4 years. Furthermore, an App is currently under development. In addition, users of the database will have the opportunity to provide feedback on the recommendations to the project team to generate further knowledge. An informed and reflected off-label use will expand the range of therapies available to patients and address the specific needs and heterogeneous requirements of this vulnerable patient group.

## Supplemental Material

sj-docx-1-pmj-10.1177_02692163251323123 – Supplemental material for Off-label drug use in palliative medicine: Delphi study for the consensus of evidence-based treatment recommendations Supplemental material, sj-docx-1-pmj-10.1177_02692163251323123 for Off-label drug use in palliative medicine: Delphi study for the consensus of evidence-based treatment recommendations by Stefanie Pügge, Aleksandra Dukic-Ott, Julian Baumgärtel, Saskia Jünger, Claudia Bausewein and Constanze Rémi in Palliative Medicine

sj-docx-2-pmj-10.1177_02692163251323123 – Supplemental material for Off-label drug use in palliative medicine: Delphi study for the consensus of evidence-based treatment recommendations Supplemental material, sj-docx-2-pmj-10.1177_02692163251323123 for Off-label drug use in palliative medicine: Delphi study for the consensus of evidence-based treatment recommendations by Stefanie Pügge, Aleksandra Dukic-Ott, Julian Baumgärtel, Saskia Jünger, Claudia Bausewein and Constanze Rémi in Palliative Medicine

## References

[1] European Medicines Agency (EMA). Guideline on good pharmacovigilance practices (GVP) Annex I – definitions (Rev 4), https://www.ema.europa.eu/en/documents/scientific-guideline/guideline-good-pharmacovigilance-practices-annex-i-definitions-rev-4_en.pdf (2017, accessed 18 March 2024).

[2] Balan S Hassali MAA Mak VSL . Two decades of off-label prescribing in children: a literature review. World J Pediatr 2018; 14: 528–540. 30218415 10.1007/s12519-018-0186-y

[bibr3-02692163251323123] Allen HC Garbe MC Lees J , et al Off-Label medication use in children, more common than we think: a systematic review of the literature. J Okla State Med Assoc 2018; 111: 776–783. 31379392 PMC6677268

[4] García-López I Cuervas-Mons Vendrell M Martín Romero I , et al Off-Label and unlicensed drugs in pediatric palliative care: a prospective observational study. J Pain Symptom Manage 2020; 60: 923–932. 32569831 10.1016/j.jpainsymman.2020.06.014

[5] Saiyed M Ong P Chew L. Off-label drug use in oncology: a systematic review of literature. J Clin Pharm Ther 2017; 42: 251–258. 28164359 10.1111/jcpt.12507

[6] Zarkavelis G Amylidi AL Verbaanderd C , et al Off-label despite high-level evidence: a clinical practice review of commonly used off-patent cancer medicines. ESMO Open 2023; 8: 100604. 36870739 10.1016/j.esmoop.2022.100604 PMC10024100

[7] Hagemann V Bausewein C Rémi C. Off-label-prescriptions in daily clinical practice – a cross-sectional national survey of palliative medicine physicians. Prog Palliat Care 2019; 27: 154–159.

[bibr8-02692163251323123] To TH Agar M Shelby-James T , et al Off-label prescribing in palliative care – a cross-sectional national survey of palliative medicine doctors. Palliat Med 2013; 27: 320–328. 23128901 10.1177/0269216312464263

[9] Kwon JH Kim MJ Bruera S , et al Off-Label medication use in the inpatient palliative care unit. J Pain Symptom Manage 2017; 54: 46–54. 28479415 10.1016/j.jpainsymman.2017.03.014 PMC5841461

[10] Wittich CM Burkle CM Lanier WL. Ten common questions (and their answers) about off-label drug use. Mayo Clin Proc 2012; 87: 982–990. 22877654 10.1016/j.mayocp.2012.04.017 PMC3538391

[11] Hagemann V Bausewein C Remi C. Drug use beyond the licence in palliative care: a systematic review and narrative synthesis. Palliat Med 2019; 33: 650–662. 31017533 10.1177/0269216319840602

[12] Hagemann V Bausewein C Remi C. Off-label use in adult palliative care – more common than expected. A retrospective chart review. Eur J Hosp Pharm 2022; 29(6): 329–335. 36283723 10.1136/ejhpharm-2020-002554 PMC9614128

[13] Rémi C Bausewein C. Zum Umgang mit off-label-use in der palliativmedizin, https://www.dgpalliativmedizin.de/images/RZ_200219_Offlabel_DS_ONLINE_aktuell_v2.pdf (2020, accessed 18 March 2024).

[14] Leitlinienprogramm Onkologie. Erweiterte S3-Leitlinie Palliativmedizin für Patienten mit einer nicht-heilbaren Krebserkrankung. Langversion 2.2 – September 2020 AWMF-Registernummer: 128/001-OL, https://www.leitlinienprogramm-onkologie.de/fileadmin/user_upload/Downloads/Leitlinien/Palliativmedizin/Version_2/LL_Palliativmedizin_Langversion_2.2.pdf (2020).

[15] Remi C Weingärtner K Hagemann V , et al Off-label drugs in palliative care: a group delphi treatment recommendation process. BMJ Support Palliat Care 2021; 11: 180–187. 10.1136/bmjspcare-2019-002165 32398226

[16] Hui D Arthur J Dalal S , et al Quality of the supportive and palliative oncology literature: a focused analysis on randomized controlled trials. Support Care Cancer 2012; 20: 1779–1785. 21935717 10.1007/s00520-011-1275-9

[17] Hui D Parsons HA Damani S , et al Quantity, design, and scope of the palliative oncology literature. Oncologist 2011; 16: 694–703. 21471275 10.1634/theoncologist.2010-0397 PMC3228194

[18] Jünger S Payne SA Brine J , et al Guidance on Conducting and REporting DElphi Studies (CREDES) in palliative care: recommendations based on a methodological systematic review. Palliat Med 2017; 31: 684–706. 28190381 10.1177/0269216317690685

[19] Pügge S Dukic-Ott A Büsel S. Neues Projekt ‘Therapieempfehlungen zum Umgang mit Off-Label-Use in der Palliativmedizin’ gestartet. Z Palliativmedizin 2021; 22: 124.

[20] Aoun SM Kristjanson LJ. Challenging the framework for evidence in palliative care research. Palliat Med 2005; 19: 461–465. 16218158 10.1191/0269216305pm1057oa

[bibr21-02692163251323123] Aoun SM Nekolaichuk C. Improving the evidence base in palliative care to inform practice and policy: thinking outside the box. J Pain Symptom Manage 2014; 48: 1222–1235. 24727305 10.1016/j.jpainsymman.2014.01.007

[22] Visser C Hadley G Wee B. Reality of evidence-based practice in palliative care. Cancer Bio Med 2015; 12: 193–200. 26487964 10.7497/j.issn.2095-3941.2015.0041 PMC4607825

[23] Black N Murphy M Lamping D , et al Consensus development methods: a review of best practice in creating clinical guidelines. J Health Serv Res Policy 1999; 4: 236–248. 10623041 10.1177/135581969900400410

[24] Biondo PD Nekolaichuk CL Stiles C , et al Applying the Delphi process to palliative care tool development: lessons learned. Support Care Cancer 2008; 16: 935–942. 17968597 10.1007/s00520-007-0348-2

[25] Niederberger M Spranger J. Delphi technique in health sciences: a map. Front Public Health 2020; 8: 457. 33072683 10.3389/fpubh.2020.00457 PMC7536299

[26] WHO. Essential medicine in palliative care: executive summary. 2013, http://www.who.int/selection_medicines/committees/expert/19/applications/PalliativeCare_8_A_R.pdf

[27] Rémi C Bausewein C Wilcock A , et al Arzneimitteltherapie in der Palliativmedizin. 4th ed. München: Elsevier Urban and Fischer, 2022.

[28] German Association of the Scientific Medical Societies (AWMF) – Standing Guidelines Commission. AWMF guidance manual and rules for guideline development, http://www.awmf.org/leitlinien/awmf-regelwerk.html (accessed 27 May 2024).

[29] Iqbal S Pipon-Young L. The Delphi method. Psychologist 2009; 22: 598–601.

[30] Deutsche Gesellschaft für Palliativmedizin. Guide to hospices and palliative care (Wegweiser Hospiz- und Palliativversorgung), https://www.wegweiser-hospiz-palliativmedizin.de/en. (2019).

[31] Limesurvey GmbH. LimeSurvey: an open source survey tool. Hamburg, Germany: LimeSurvey GmbH.

[32] von der Gracht HA . Consensus measurement in Delphi studies: review and implications for future quality assurance. Technol Forecast Soc Change 2012; 79: 1525–1536.

[33] Nasa P Jain R Juneja D. Delphi methodology in healthcare research: how to decide its appropriateness. World J Methodol 2021; 11: 116–129. 34322364 10.5662/wjm.v11.i4.116 PMC8299905

[34] Stewart D Gibson-Smith K Maclure K , et al A modified Delphi study to determine the level of consensus across the European Union on the structures, processes and desired outcomes of the management of polypharmacy in older people. PLoS One 2017; 12: e0188348. 10.1371/journal.pone.0188348 PMC5695766 29155870

[35] Toscani F. Prescribing in palliative care: a quest for appropriateness. Palliat Med 2013; 27: 293–294. 23538673 10.1177/0269216313479843

[36] Mann N-K Mathes T Sönnichsen A , et al Potenziell inadäquate Medikation für ältere Menschen: PRISCUS 2.0. Dtsch Arztebl Int 2023; 120: 3–10. 36507719 10.3238/arztebl.m2022.0377 PMC10035347

[bibr37-02692163251323123] Pazan F Weiss C Wehling M , et al The EURO-FORTA (Fit fOR The Aged) list version 2: consensus validation of a clinical tool for improved pharmacotherapy in older adults. Drugs Aging 2023; 40: 417–426. 37129833 10.1007/s40266-023-01024-6 PMC10152014

[38] Schol J Wauters L Dickman R , et al United European Gastroenterology (UEG) and European Society for Neurogastroenterology and Motility (ESNM) consensus on gastroparesis. United European Gastroenterol J 2021; 9(3): 287–306. 10.1002/ueg2.12060 PMC8259275 33939892

[39] Riva A Amadori E Vari MS , et al Impact and management of drooling in children with neurological disorders: an Italian Delphi consensus. Ital J Pediatr 2022; 48: 118. 35854335 10.1186/s13052-022-01312-8 PMC9297577

[bibr40-02692163251323123] Ostgathe C Bausewein C Schildmann E , et al Expert-approved best practice recommendations on the use of sedative drugs and intentional sedation in specialist palliative care (SedPall). BMC Palliat Care 2023; 22(1): 126. 37667303 10.1186/s12904-023-01243-z PMC10476406

[41] Bilgin ES Ülgüt R Schneider N , et al Improving primary palliative care – a delphi consensus study on measures for general practice in Germany. BMC Prim Care 2022; 23: 12. 35172733 10.1186/s12875-021-01613-7 PMC8762944

[42] Niederberger M Deckert S. The Delphi technique: methodology, variants and usage examples. Z Evid Fortbild Qual Gesundheitswes 2022; 174: 11–19. 10.1016/j.zefq.2022.08.007 36137932

[43] van der Zanden TM Smeets NJL de Hoop-Sommen M , et al Off-label, but on-evidence? A review of the level of evidence for pediatric pharmacotherapy. Clin Pharmacol Ther 2022; 112: 1243–1253. 36069288 10.1002/cpt.2736 PMC9828396

[44] Häder M. Delphi-Befragungen: Ein Arbeitsbuch. 3rd ed. Wiesbaden: Springer VS Wiesbaden, 2014.

[45] Niederberger M Renn O. Das Gruppendelphi-Verfahren. Vom Konzept bis zur Anwendung. Wiesbaden: Springer Fachmedien Wiesbaden GmbH, 2018.

