# Supplementary material for: Off-label drug use in palliative medicine: Delphi study for the consensus of evidence-based treatment recommendations
Source: Palliat Med. 2025 Mar 15;39(5):530–42. doi: 10.1177/02692163251323123 (PMC12033383; doi:10.1177/02692163251323123)
Supplement: sj-docx-1-pmj-10.1177_02692163251323123 – Supplemental material for Off-label drug use in palliative medicine: Delphi study for the consensus of evidence-based treatment recommendations [file sj-docx-1-pmj-10.1177_02692163251323123.docx]

| **Gastroparesis** | | | | | | | | | |
| --- | --- | --- | --- | --- | --- | --- | --- | --- | --- |
| Clonidine | Gastroparesis | Clonidine CAN be used to improve the symptoms of diabetic gastroparesis if better documented treatment options are not effective or are not an option, subject to careful risk-benefit assessment and close monitoring of side effects. **LoE/RG 1- / 0** | 60 | 88.5 | 2 | 0 | 43.3 | 2.2 |  |
| Clonidine | Gastroparesis | Clonidine SHALL NOT be used to treat the symptoms of gastroparesis of other causes, as there are better documented therapeutic options. **LoE / RG 4 / A** | 75 | 86.8 | 2 | 1 | 50.7 | 2.1 |  |
| Domperidone | Gastroparesis | Domperidone SHOULD be used for the symptomatic treatment of gastroparesis if dietary measures are not sufficient and metoclopramide is not effective or not suitable for symptom control. **LoE / RG 1- / B** | 75 | 93.7 | 2 | 1 | 84.0 | 2.1 |  |
| Erythromycin | Gastroparesis | Erythromycin CAN be used to improve symptoms of gastroparesis if dietary measures and first-line prokinetics such as metoclopramide, domperidone or prucalopride are not effective or are not an option. **LoE / RG 2++ / 0** | 75 | 84.3 | 2 | 1 | 68.0 | 2.1 |  |
| Mirtazapine | Gastroparesis | Mirtazapine CAN be used to improve the symptoms of gastroparesis if dietary measures and first-line prokinetics such as metoclopramide, domperidone or prucalopride are not effective or are not an option. **LoE / RG 2++ / 0** | 75 | 84.6 | 2 | 0 | 53.3 | 2.1 |  |
| **Hot flushes** | | | | | | | | | |
| Clonidine | Hot flushes | Clonidine CAN be used for the treatment of hot flushes in women with a history of breast cancer and in postmenopausal women who refuse hormonal therapy or for whom it is not an option, taking into account the side effects and possible alternative therapies. **LoE / RG 1+ / 0** | 67 | 92.1 | 2 | 0 | 56.7 | 1.2 |  |
| Clonidine | Hot flushes | Clonidine SHALL NOT be used for the treatment of hot flushes in men after prostate cancer. **LoE / RG 1+ / A** | 64 | 100.0 | 2 | 1 | 60.9 | 1.1 |  |
| Levetiracetam | Hot flushes | Levetiracetam CAN be used for the treatment of distressing hot flushes in patients with a history of breast cancer or in patients who refuse treatment with oestrogens. **LoE / RG 2+ / 0** | 64 | 83.3 | 2 | 0 | 56.3 | 1.1 |  |
| **Hiccups** | | | | | | | | |  |
| Baclofen | Hiccups | Baclofen SHOULD be considered in the treatment of persistent hiccups for central suppression of the hiccup reflex. **LoE / RG 1- / B** | 64 | 96.3 | 2 | 1 | 84.4 | 1.1 | |
| Clonidine | Hiccups | Clonidine SHALL NOT be used for the treatment of hiccups, as more effective therapeutic alternatives have been described. **LoE / RG 3 / A** | 64 | 92.2 | 1 | 1 | 79.7 | 1.1 | |
| Dexamethasone | Hiccups | Dexamethasone SHOULD NOT be used for the symptomatic treatment of hiccups, as there are better proven treatment options for this and it can also trigger hiccups itself. **LoE / RG 3 / B** | 69 | 90.8 | 2 | 1 | 94.2 | 1.2 | |
| Erythromycin | Hiccups | Erythromycin SHOULD NOT be used for therapy refractory hiccups, as there are no data available in the literature. there is a risk of bacterial resistance and there are also better documented alternatives with fewer side effects**. LoE / RG 4 / B** | 75 | 96.7 | 1 | 1 | 80.0 | 2.1 | |
| Gabapentin | Hiccups | Gabapentin CAN be used in patients with refractory hiccups if other measures have failed and first-line therapies such as baclofen or metoclopramide are not effective or are not an option. **LoE / RG 2++ / 0** | 75 | 98.4 | 2 | 1 | 82.7 | 2.1 | |
| Haloperidol | Hiccups | Haloperidol CAN be used in the drug treatment of therapy refractory hiccups to centrally suppress the hiccup reflex if treatment with baclofen, gabapentin or metoclopramide is not sufficiently effective. **LoE / RG 3 / 0** | 64 | 92.6 | 2 | 1 | 84.4 | 1.1 | |
| Metoclopramide | Hiccups | Metoclopramide SHOULD be considered for the treatment of persistent hiccups with gastric or peripheral causes. **LoE / RG 1- / B** | 64 | 94.6 | 1 | 1 | 87.5 | 1.1 | |
| Midazolam | Hiccups | Midazolam CAN be used in the last days of life to treat hiccups if other measures are not effective or not sufficiently effective, especially if sedation is desired at the same time. **LoE / RG 3 / 0** | 69 | 100.0 | 2 | 1 | 91.3 | 1.2 | |
| Pregabalin | Hiccups | Pregabalin CAN be used in patients with therapy refractory hiccups if other measures have failed and first-line therapies such as baclofen or metoclopramide and gabapentin are not effective or are not an option. **LoE / RG 3 / 0** | 75 | 96.4 | 2 | 1 | 73.3 | 2.1 | |

Supplement: Further consented treatment recommendations of the first two Delphi studies (n=participants in the respective Delphi procedure; consensus: % agreement of very certain/slightly certain answers; certainty: % votes with very certain/slightly certain; IQR=interquartile range; DS=consensus achieved in Delphi study and round X.Y)
