# Supplementary material for: Off-label drug use in palliative medicine: Delphi study for the consensus of evidence-based treatment recommendations
Source: Palliat Med. 2025 Mar 15;39(5):530–42. doi: 10.1177/02692163251323123 (PMC12033383; doi:10.1177/02692163251323123)
Supplement: sj-docx-2-pmj-10.1177_02692163251323123 – Supplemental material for Off-label drug use in palliative medicine: Delphi study for the consensus of evidence-based treatment recommendations [file sj-docx-2-pmj-10.1177_02692163251323123.docx]

| **DS** | **Drug** | **OLU** | **original recommendation** | **consensus [%]** | **DS** | **Comments/rephrased recommendation** | **consensus [%]** |
| --- | --- | --- | --- | --- | --- | --- | --- |
| 1.1. | Gabapentin | Pruritus | Gabapentin CAN be considered for opioid-induced pruritus.  LoE / RG 2- / 0 | 79,2 |  | *postponed to a future Delphi process for content-related reasons* |  |
| 1.1. | Midazolam | Pruritus | Midazolam CAN be considered for the prevention of opioid-induced pruritus. LoE / RG 1- / 0 | 53,5 |  | *postponed to a future Delphi process for content-related reasons* |  |
| 1.1. | Dexamethasone | Subcutaneous application | Continuous subcutaneous infusion of dexamethasone CAN be considered if other routes of administration and subcutaneous bolus administration are not suitable. LoE / RG 1- / 0 | 77,2 | 1.2. | Continuous subcutaneous infusion of dexamethasone SHOULD NOT be used. LoE / RG 1- / B | 97,0 |
| 1.1. | Dexamethasone | Subcutaneous application | The prophylactic addition of dexamethasone to infusion solutions for continuous subcutaneous infusion CAN be considered if the mixtures present an increased risk of irritation. To avoid incompatibilities Dexamethason SHALL be added last to the mixture. LoE / RG 2- / 0 | 58,3 | 1.2. | The prophylactic addition of dexamethasone to infusion solutions for continuous subcutaneous infusion CAN be considered if the mixtures present an increased risk of skin irritation. To avoid incompatibilities Dexamethason SHALL be added last to the mixture. LoE / RG 2- / 0 | 72,2 |
| 1.1. | Haloperidol | Subcutaneous application | Haloperidol SHALL be administered subcutaneously if oral administration is contraindicated or difficult (e.g. in cases of dysphagia) or if absorption of the active ingredient is impaired after oral administration (e.g. in cases of vomiting) or if no other alternative routes of administration are possible. LoE / RG 2+ / A | 95,1 | 1.2. | Haloperidol SHOULD be administered subcutaneously as a short infusion if oral administration is contraindicated or difficult (e.g. in cases of dysphagia) or if absorption of the active ingredient is impaired after oral administration (e.g. in cases of vomiting) or if no other alternative routes of administration are possible. LoE / RG 2+ / B | 90,5 |
| 1.1. | Haloperidol | Subcutaneous application | Haloperidol SHALL be administered as a continuous subcutaneous infusion if oral administration is contraindicated or difficult (e.g. in cases of dysphagia) or if absorption of the active ingredient is impaired after oral administration (e.g. in cases of vomiting) or if no other alternative routes of administration are possible. LoE / RG 2+ / A | 76,8 | 1.2. | Haloperidol CAN be administered as a continuous subcutaneous infusion if oral administration is contraindicated or difficult (e.g. in cases of dysphagia) or if absorption of the active ingredient is impaired after oral administration (e.g. in cases of vomiting) or if no other alternative routes of administration are possible and short subcutaneous infusion is less suitable. LoE / RG 2+ / 0 | 82,8 |
| 1.1. | Midazolam | Subcutaneous application | Subcutaneous administration of midazolam SHOULD be considered when other authorised routes of administration have failed.  LoE / RG 2+ / B | 96,8 | 1.2. | Subcutaneous administration of midazolam SHOULD be considered when other authorised routes of administration have failed or are unsuitable. LoE / RG 2+ / B | 97,0 |
| 1.1. | Clonidine | Ascites | Clonidine CAN be considered for patients with refractory ascites due to liver cirrhosis who do not respond to standard therapy consisting of a salt-reduced diet, spironolactone and furosemide.  LoE / RG 2 / 0 | 68,8 | 1.2. | *Added in the background text to the therapy recommendation: Clonidine promotes the excretion of fluid via the kidneys by inhibiting the renin-angiotensin-aldosterone system and the sympathetic nervous system.* | 91,4 |
| 1.1. | Clonidine | Hot flushes | Clonidine CAN be used for the treatment of hot flushes in women with a history of breast cancer and in postmenopausal women, taking into account the side effects and possible alternative therapies. LoE / RG 1+ / 0 | 71,4 | 1.2. | Clonidine CAN be used for the treatment of hot flushes in women with a history of breast cancer and in postmenopausal women who refuse hormonal therapy or for whom it is not an option, taking into account the side effects and possible alternative therapies. LoE / RG 1+ / 0 | 92,1 |
| 2.1. | Pregabalin | Rectal application | Rectal administration of pregabalin CAN be considered if oral administration is not possible or other medications are not sufficient to control symptoms. LoE / RG 3 / 0 | 69,9 | 2.2. | *Change in background text to treatment recommendation: consideration of additional evidence (submitted manuscript and research snapshot)* | 96,1 |
| 2.1. | Erythromycin | Bronchorrhoea | Erythromycin CAN be considered for symptomatic therapy in patients with adenocarcinoma and bronchorrhoea. LoE (BoE)/RG: 2++(3)/0 | 41,9 | 2.2. | Erythromycin CAN be considered for symptomatic therapy in patients with adenocarcinoma and bronchorrhoea if antitumour therapy with gefitinib or erlotinib is not indicated or does not lead to any improvement and other off-label alternatives are not feasible or show no effect after individual benefit assessment. LoE (BoE)/RG: 2++(3)/0 | 71,0 |
| 2.1. | Clonidine | Gastroparesis | Clonidine CAN be used to improve the symptoms of diabetic gastroparesis if better documented treatment options are not effective or are not an option. LoE / RG 1- / 0 | 73,3 | 2.2. | Clonidine CAN be used to improve the symptoms of diabetic gastroparesis if better documented treatment options are not effective or are not an option, subject to careful risk-benefit assessment and close monitoring of side effects. LoE/RG 1-/0 | 88,5 |

*SUPPLEMENT: Changes in treatment recommendations in the 1st and 2nd round of the Delphi studies (marked in red) (DS= Delphi study and round; LoE=Level of Evidence; RG= recommendation grade)*
